# A Window of Opportunity: Perilymph Sampling from the Round Window Membrane Can Advance Inner Ear Diagnostics and Therapeutics

**DOI:** 10.3390/jcm11020316

**Published:** 2022-01-09

**Authors:** Madeleine St. Peter, Athanasia Warnecke, Hinrich Staecker

**Affiliations:** 1 Department of Otolaryngology-Head & Neck Surgery, University of Kansas Medical Center, Kansas City, KS 66160, USA; mstpeter@kumc.edu; 2 Department of Otolaryngology Head and Neck Surgery, Hannover Medical School, D-30625 Hanover, Germany; warnecke.athanasia@mh-hannover.de

**Keywords:** perilymph, round window, stapedectomy, cochlear implantation, sensorineural hearing loss

## Abstract

In the clinical setting, the pathophysiology of sensorineural hearing loss is poorly defined and there are currently no diagnostic tests available to differentiate between subtypes. This often leaves patients with generalized treatment options such as steroids, hearing aids, or cochlear implantation. The gold standard for localizing disease is direct biopsy or imaging of the affected tissue; however, the inaccessibility and fragility of the cochlea make these techniques difficult. Thus, the establishment of an indirect biopsy, a sampling of inner fluids, is needed to advance inner ear diagnostics and allow for the development of novel therapeutics for inner ear disease. A promising source is perilymph, an inner ear liquid that bathes multiple structures critical to sound transduction. Intraoperative perilymph sampling via the round window membrane of the cochlea has been successfully used to profile the proteome, metabolome, and transcriptome of the inner ear and is a potential source of biomarker discovery. Despite its potential to provide insight into inner ear pathologies, human perilymph sampling continues to be controversial and is currently performed only in conjunction with a planned procedure where the inner ear is opened. Here, we review the safety of procedures in which the inner ear is opened, highlight studies where perilymph analysis has advanced our knowledge of inner ear diseases, and finally propose that perilymph sampling could be done as a stand-alone procedure, thereby advancing our ability to accurately classify sensorineural hearing loss.

## 1. Introduction

Permanent hearing loss affects more than 5% of the world’s population and is the most common sensory deficit in developed countries [1]. It can be divided into two categories: conductive hearing loss (CHL) and sensorineural hearing loss (SNHL). SNHL makes up 90% of the cases and can be caused by damage to any of the more than 20 inner ear cell types responsible for hearing [2,3,4]. As with all other tissues in the body, damage to the inner ear can be caused by genetic, infectious, inflammatory, toxic, or degenerative mechanisms. At present, SNHL is defined by the hearing threshold (i.e., mild, moderate, severe, severe to profound, and profound) combined with clinical features (progressive, sudden, fluctuating, etc.). The degree of hearing loss is determined by pure tone audiometry, which is a subjective patient response-based test. Objective auditory diagnostic testing such as otoacoustic emissions can localize hearing loss to a decline in outer hair cell function but is ineffective in more severe hearing losses [5]. Without accurate localization of disease and disease mechanisms, only rehabilitative treatment options such as hearing aids or cochlear implants can be offered. Those with severe or profound SNHL who identify 50% or fewer words on key word testing can be offered cochlear implantation (CI), which can have variable outcomes.

The location and fragility of the cochlea poses a significant diagnostic challenge. The cochlea is buried deep within the temporal bone and is surrounded by a thick bony otic capsule, making it difficult to access for diagnostic profiling [6]. Violation of the tight junction system that separates the scala media (endolymph compartment) from the remainder of the inner ear results in complete loss of residual hearing, making it impossible to biopsy the inner ear. While postmortem and animal studies of the cochlea have offered invaluable insights, we still have a limited understanding of what is occurring on a molecular level in patients with active inner ear disease [7]. The successful development of hearing preservation cochlear implantation raises the possibility that perilymph in the scala tympani of the inner ear can be accessed without loss of residual hearing [8,9].

Perilymph is found in the scalae tympani, scala vestibuli, and in the balance portions of the inner ear and is similar in composition to cerebral spinal fluid (CSF). It bathes multiple structures necessary for signal transduction including spiral ganglion cell bodies, the auditory nerve, the hair cells, as well as portions of the lateral wall of the cochlea and is critical to sound transmission within the cochlea [10,11]. Perilymph can potentially be sampled during inner ear surgery such as stapedectomy, labyrinthectomy, and cochlear implantation, which allows for comparison between patient subpopulations with SNHL and CHL [12,13]. Since the advent of the sampling technique in the mid-1900s, human and animal studies have revealed proteins, metabolites, and microRNAs (miRNAs) specific to subtypes of SNHL in perilymph. In addition, perilymph profiles have been correlated to surgical outcomes and prognosis, specifically patient response to cochlear implantation (CI). We propose that perilymph sampling via the round window membrane (RWM) can be developed as a safe outpatient procedure and can serve as a “liquid biopsy” to guide diagnosis and treatment of SNHL. 

## 2. History of Human Perilymph Sampling

### 2.1. Postmortem Profiling

Many perilymph profiling studies in humans have been performed using postmortem samples. These studies largely form the backbone of our current understanding of the chemical and protein makeup of human perilymph. In fact, postmortem studies were the first to accurately describe ionic concentrations of human perilymph [14,15] and investigate changes in ionic and protein compositions associated with inner ear diseases such as otosclerosis [16,17,18]. 

Postmortem perilymph sampling has also allowed for comparisons between CSF, serum, and perilymph, furthering our understanding of inner ear anatomy and the role of each fluid in hearing. Arrer et al. used proteome analysis to compare the levels of α1-antitrypsin and pre-albumin in the CSF, perilymph, and serum. Each fluid demonstrated a different pattern, suggesting that they are distinct fluids with different roles in the inner ear [19]. Later, Palva et al. used postmortem samples to compare the esterase composition of endolymph, perilymph, serum, and CSF. This study revealed distinct patterns of esterase concentration of endolymph and perilymph compared to serum and CSF, further suggesting that these are all separate fluids [17]. More recently, postmortem analysis of perilymph has been used to catalog the presence of miRNA in the cochlea. For example, over 500 miRNAs have been detected in the endolymph and perilymph of postmortem samples, with 481 differentially expressed in patients with a vestibular disorder called benign paroxysmal positional vertigo [20]. 

### 2.2. Intraoperative Sampling in Humans

Although many advances have been made using postmortem sampling, molecular analysis in these studies is limited because of rapid autolysis and degradation of DNA, RNA, and proteins [21]. To overcome this, human perilymph can be sampled from living subjects intraoperatively. Sampling during open ear surgeries dates back as far as 1950, when Waltner et al. extracted perilymph from the semicircular canal of patients with Meniere’s disease (SNHL) during labyrinthectomy to compare perilymph and endolymph compositions [11]. Stapedectomy was the first surgical procedure in which the inner ear was routinely opened and, until the advent of cochlear implantation, yielded the bulk of data on perilymph composition. 

Intraoperative perilymph sampling can also be used to assess direct and systemic drug delivery to the inner ear. Molecules perfused into a perilymph compartment (ideally the scala tympani) have direct access to the cells of the inner ear; therefore, optimal drug delivery to the inner ear depends on high concentrations in perilymph. Most studies have focused on quantifying steroid delivery specifically, given that intratympanic and intravenous steroids are commonly used as first line for certain subtypes of SNHL. Using intraoperative sampling during CI, it was found that inner ear levels are dependent on the systemic intravenous steroid dose received [22], and that intratympanic injections result in much higher drug delivery when compared to intravenous infusion [23].

## 3. Animal Models of Sensorineural Hearing Loss and Perilymph Sampling

### 3.1. Noise-Induced Hearing Loss

Noise-induced hearing loss (NIHL) accounts for one-third of all acquired cases of SNHL. Exposure to noises greater than 90 decibels for prolonged periods can cause mitochondrial pathology, excessive excitatory neurotransmitter release, and reduced blood flow to the cochlea, all of which result in the production of reactive oxygen species (ROS). ROS then inflict oxidative damage to the cochlear structures necessary for hearing [24]. 

Perilymph sampling in animal models has been used to measure changes in ROS, cytokines, and other metabolites in the cochlea before and after noise exposure. In mice, one hour of exposure to 110 dB causes a fourfold increase in hydroxy radical concentration in perilymph [25]. In guinea pigs, impulse noise exposure with subsequent perilymph analysis resulted in the discovery of seven altered metabolites following noise exposure [26]. Furthermore, inhalation of hydrogen gas was protective from ROS-mediated NIHL, and metabolic changes associated with hydrogen gas administration were reflected in perilymph [26]. Changes in cytokines such as tumor necrosis factor and interleukin 6 have also been detected after noise exposure using perilymph sampling in mice [20]. Although not currently relevant for understanding human disease, these findings suggest that profiling of ROS species and cytokines in human perilymph may lead to a further understanding of the mechanisms of NIHL as well as other subtypes of SNHL that may involve the ROS or inflammatory pathway.

### 3.2. Age-Related Hearing Loss

Age-related hearing loss (ARHL), also called presbycusis, is the most common subtype of SNHL and has a multifactorial etiology. It was hypothesized by Harman in 1956 that environmental exposures such as noise, ototoxic substances, and age-related cochlear hypoperfusion increase oxidative stress and contribute to the development of ARHL in those with genetic predisposition [27,28]. Though there are multiple animal models of ARHL and several lines of evidence supporting Harman’s free radical theory in animals, there is currently a paucity of literature regarding animal models of perilymph profiles in ARHL. 

### 3.3. Perilymph Expression Patterns across Species

Intraoperative perilymph sampling allows for direct comparison of human and animal tissues. It can therefore be used to identify feasible drug targets and guide animal model development. Although there is limited literature comparing animal and human perilymph, there are some studies that compare protein profiles. In 2011, Lysaght et al. found that perilymph from patients undergoing CI for SNHL had 31 orthologs in common with the mouse perilymph profile collected by Swan et al. in 2009 [29,30,31]. Comparison of human perilymph to guinea pigs has revealed a 64% overlap in protein profile, with apolipoproteins, enzymes, and immunoglobulins among the highly conserved classes [32]. This suggests that although useful, animal models are not a complete substitute for human data.

## 4. Human Perilymph Proteomics

### 4.1. Perilymph Proteins Specific to Subtypes of SNHL

Protein analysis of perilymph has elucidated the pathways involved in SNHL and has allowed for comparisons between subtypes of SNHL. Mass spectrometry has been used to create and compare comprehensive libraries of perilymph proteins in CI and vestibular schwannoma (VS) patients [31]. Although both patient groups have SNHL, comparison of their perilymph revealed differentially expressed proteins in VS samples including m-crystallin and LDL receptor related protein 2, which serve as the first potential markers of VS. Later studies by Rasmussen et al. discovered a protein that correlates with degree of tumor-associated hearing loss in VS perilymph: alpha-2-HS-glycoprotein [33]. In a large-scale analysis of perilymph from CI patients, proteins specific to infectious and congenital causes of SNHL were identified. In this same study, 97 proteins were found to be present only in adults with idiopathic SNHL when compared to children with idiopathic SNHL, revealing proteins potentially implicated in presbycusis [13,33]. 

Proteins specific to Meniere’s disease (SNHL) have also been identified in human perilymph using liquid chromatography with tandem mass spectrometry. In 2019, Lin et al. compared the perilymph of Meniere’s disease patients to the perilymph of normal hearing patients with skull-base meningiomas and discovered 38 proteins with differential abundance [29], four of which have known roles in the pathogenesis of Meniere’s disease. Additional groups have used protein analysis to investigate entire pathways and protein families altered in patients with subtypes of SNHL, including inflammatory pathways, heat shock proteins, and neurotrophin pathways.

### 4.2. Inflammatory Pathways

Some patients with progressive or sudden SNHL are responsive to intratympanic injection of anti-inflammatory medications such as steroids. Therefore, identifying inflammatory protein mediators in the inner ear is of interest to researchers [34]. Warnecke et al. used perilymph from CI patients to perform protein multiplex analysis, which resulted in the discovery of key inflammatory mediators and potential drug targets for SNHL. In addition, protein patterns were correlated with residual hearing pre-CI [35]. Some of the proteins discovered in this study were also the targets of drugs with demonstrated efficacy in animal models. For instance, insulin-like growth factor binding protein 1 (IGFBP1), a known regulator of insulin-like growth factor 1 (IGF-1), was highly expressed in the perilymph of patients with complete loss of auditory function when compared to patients with residual hearing. Animal studies have shown that applying recombinant IGF-1 to the RWM protects against noise-induced hearing loss in guinea pigs and rats [35,36]. Furthermore, in a human trial, treating patients with sudden SNHL refractory to systemic steroids with middle ear IGF-1 was superior to intratympanic steroid injections [37]. This demonstrates that perilymph analysis and animal studies can be linked to potentially develop novel diagnostic and therapeutic interventions.

### 4.3. Neurotrophin Pathway

Studies of the mouse cochlea in vivo have demonstrated that neurotrophic factors such as brain-derived neurotrophic factor (BDNF) and neurotrophin 3 (NT-3) are essential to neuron survival, and application of these factors can rescue and protect spiral ganglia neurons, which are critical for signal transduction and hearing [38,39,40]. Consistent with these findings, human perilymph protein analysis revealed that higher levels of BDNF-regulated proteins are correlated to the presence of residual hearing prior to implantation and to better cochlear implant performance. Conversely, decreased levels of BDNF-regulated proteins were associated with profoundly deaf patients versus those with residual hearing [41].

### 4.4. Heat Shock Proteins

Heat shock proteins (HSPs) are thought to protect tissues by refolding denatured or misfolded proteins. They have been implicated in many disease processes, and recently in the pathogenesis of sudden SNHL, Meniere’s disease, and idiopathic SNHL. Serum values of HSPs are significantly higher in those with sudden SNHL compared to normal hearing controls [42]. Additionally, antibodies to HSPs are present in patients with Meniere’s disease and levels correlate with disease activity [43]. In human perilymph, 10 subgroups of HSPs have been identified, with higher levels present in patients with residual hearing before undergoing CI and VS removal [44].

## 5. Human Perilymph Metabolome and Transcriptome

### 5.1. Perilymph Metabolome

Characterizing the metabolic composition of perilymph fluid is critical for understanding the pathophysiology of deafness and predicting surgical outcomes. This is especially critical for CI patients, as the metabolites in perilymph interact directly with the electrode placed in the cochlea. Perilymph proteome analysis has revealed a relationship between the levels of metabolites such as N-acetylneuraminate, glutaric acid, cystine, 2-methylpropanoate, butanoate, and xanthine and the duration of SNHL [45]. Although there have been mixed results, there is some evidence that those with a longer duration of SNHL have decreased speech comprehension post-CI [46], which suggests that a RWM perilymph “tap” with metabolic analysis might be useful for predicting speech comprehension after CI. Although further studies are needed to confirm this theory, it is a promising potential tool to determine candidacy for cochlear implantation.

Similar to rat, guinea pig, and mouse models, humans with profound SNHL from multiple etiologies display characteristic metabolites in perilymph. In one human study, the perilymph of patients with profound SNHL was compared to that of those with otosclerosis (CHL). Those with profound SNHL had significantly higher superoxide in perilymph and were positive for the ROS-producing enzyme xanthine [47].

### 5.2. MicroRNAs as Biomarkers for SNHL

MiRNAs are small non-coding RNAs that can be found in body fluids. They serve as reliable biomarkers and prognostic indicators for multiple other neurodegenerative diseases, including Alzheimer’s, amyotrophic lateral sclerosis, and Parkinson’s [48,49,50]. They have also recently been used to differentiate subtypes of SNHL and predict prognosis. In 2018, Shew et al. demonstrated that machine learning algorithms can use miRNA perilymph profiles to delineate between SNHL and CHL and can predict residual hearing after CI with 100% accuracy. In addition, comparison of miRNA profiles between patients with otosclerosis (CHL) and Meniere’s disease (SNHL) using microarray has revealed miRNAs differentially expressed in the perilymph of patients with Meniere’s disease [51,52]. As shown in recent proteomics studies, miRNA expression may also predict the status of neurotrophin signaling in cochlear implant patients [53]. 

In summary, we can already derive a wealth of information from a sample of perilymph, including predictors of hearing loss, markers of a pro-inflammatory state, factors predicted to protect the inner ear during surgery, and potential predictors of cochlear implant outcomes. Identifying the mechanisms of hearing loss in real time will also allow optimized pharmacologic intervention. To take advantage of this wealth of data, this information needs to be available prior to initiating a planned treatment such as CI. 

## 6. Applications of Human Perilymph Sampling

Opening the inner ear was historically considered impossible due to the risk of hearing loss; however, recent experience with hearing preservation cochlear implantation and past sampling studies on stapedectomy patients indicates that it is possible to manipulate the inner ear with no or minimal loss of residual hearing in most patients [54]. We propose that perilymph can be sampled as a stand-alone procedure. Several different existing ear surgeries can be used to model the development of a perilymph sampling procedure including cochlear implantation, stapedectomy, and cochleosacculotomy. Initial applications of perilymph sampling will likely be in enhancing CI and evaluating progressive hearing loss. As more targeted therapeutics for hearing loss emerge, further applications will develop [55]. 

### 6.1. Cochlear Implantation 

A cochlear implant is a prosthetic device inserted into the inner ear of patients with severe SNHL and poor speech perception who have minimal improvement with the use of hearing aids. Criteria for undergoing implantation have been recently expanded from including only patients with profound hearing loss to those patients with significant residual hearing but poor speech understanding. The surgery is performed by drilling a mastoidectomy and then entering the middle ear space through a posterior tympanotomy (the space between the incus, chorda tympani, and facial nerves) [56]. The bony overhang of the round window is then drilled down to visualize the RWM. An incision is made in the RWM, allowing for the sampling of a small amount of perilymph. The electrode can then be advanced through the window. 

CI can improve the speech perception ability in 82.0% of adults with post lingual hearing loss and 53.4% of adults with prelingual hearing loss and can markedly improve quality of life in the responders [57]. However, there is a proportion of patients who do not benefit, and most continue to lose hearing that was present before the operation [55]. Unfortunately, clinicians currently have no way to predict which patients will respond to CI. Although duration of hearing loss and pre-implantation speech perception are thought to be correlated to outcomes, studies have shown mixed results in small sample sizes, and there is still no consensus regarding which patient factors predict functional hearing over time [58]. Characteristics such as sex and age also have mixed results regarding correlation to residual hearing, and do not account for the large variability in patient response to CI [55]. In animal models, some groups have shown that trauma during surgery can induce inflammation and affect post-op hearing [59]; however, human studies have shown that hearing continues to decline long after post-operative inflammation has resolved [55]. Taken together, this has led to the hypothesis that etiology of SNHL rather than patient profile or surgical factors may have the most influence on CI outcomes, further demonstrating the need for subclassification of SNHL. 

Most of the current perilymph sampling studies have focused on perilymph sampled from cochlear implant patients and information derived from these studies may yield information on optimal pharmacologic intervention to protect hearing during the implantation process. Sampling could also help predict who would be a candidate for supplementation with neurotrophins or who could benefit from drug eluting cochlear implant electrodes [60]. Since patients with significant residual hearing are being successfully implanted, perilymph sampling at the time of implantation can give us initial safety data on the procedure when it is performed at the same time as cochlear implantation. Hannover Medical School has been routinely sampling perilymph on all implant patients and has not seen a decline in their hearing preservation rates [13,61]. However, safety data should not be gleaned solely from CI procedures. Both the perilymph sampling procedure and CI electrode insertion require puncture of the RWM, which causes perilymph egress. Therefore, if these are done simultaneously, it will be difficult to draw conclusions regarding the safety of perilymph sampling specifically.

### 6.2. Stapedectomy and Cochleosacculotomy

Several other operations access the inner ear fluid spaces. Stapedectomy is commonly performed for patients with otosclerosis, a cause of CHL. Stapedectomy is well tolerated and significantly improves hearing, with some studies showing up to 70% of patients achieving an air–bone gap of 20 dB or better [62,63]. 

Although opening the stapes footplate accesses the perilymphatic spaces of the inner ear, this approach would probably not be applicable in routine perilymph sampling in patients without a fixed stapes footplate. Additionally, in patients undergoing workup for SNHL, the stapes supra-structure would impede access to the inner ear. Access to the middle ear, which contains the stapes bone, is gained by making an incision in the auditory canal and lifting the tympanic membrane. The bony scutum is then shaved down, allowing for the visualization of multiple middle and inner ear structures, including the round window niche. 

Stapedectomy is generally not indicated for SNHL and is therefore not useful for directly profiling patients with SNHL. However, it is commonly performed for CHL, which provides an opportunity for a control group. Although sampling during stapedectomy is a debated topic among some clinicians, multiple groups have reported safety using this methodology, and stapedectomy has yielded valuable information on pathogenesis of CHL [18,22,46,64,65,66,67,68,69,70,71,72,73,74,75]. In this technique, perilymph is not collected from the stapedotomy opening, but from the surrounding footplate where perilymph has already egressed out of the vestibule. Therefore, it is unlikely that collection of perilymph is significantly altering post-operative outcomes.

The RWM itself is accessed during another open ear surgery called cochleosacculotomy, indicated for patients with refractory MD (SNHL) who have minimal or no residual hearing in the affected ear [76]. In this procedure, the middle ear is opened and the bony overhang of the round window niche is removed. A 4 mm right angle pick is then used to obliterate the inner ear. A similar approach to the round window could be used to develop a non-destructive sampling of inner ear fluid.

### 6.3. Proposed Method of RWM “Tap”

**Figure 1:**
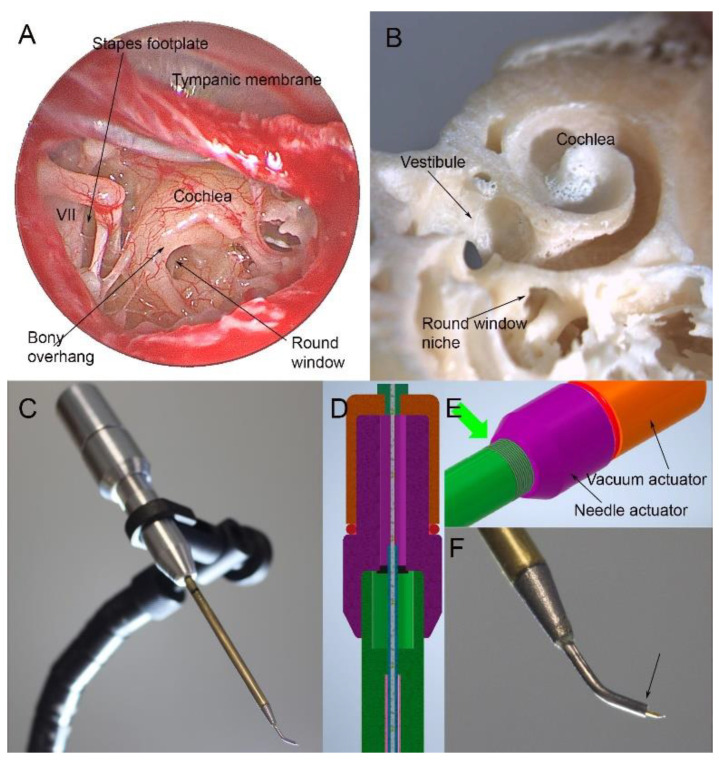
Endoscopic view of the right human middle ear (**A**). After lifting the tympanic membrane, the round window can be seen but is partially obscured by a bony overhang. Only a small area of the stapes footplate is visible next to the facial nerve (VII), making it difficult to access. The anatomy of the cochlea can be seen in a human temporal bone in which the cochlea has been opened (**B**). The round window niche allows access to the basal turn of the cochlea. A prototype sampling device features a 56 mm long shaft that can be passed down the ear canal to reach the round window (**C**). The device has two internal actuators, one advancing a needle and one allowing a plunger to be withdrawn from the needle/internal reservoir (**D**,**E**) through threading built into the device (green arrow, (**E**)). This allows advancement of a needle from the curved tip of the device in submillimeter increments and withdrawal of up to 10 µL of fluid. The tip of the device is shown in (**F**) and measures 0.86 mm at the tip (arrow) from which the needle is deployed.

For sampling perilymph, a standard transcanal approach would be used. After making a cut in the ear canal skin, the tympanic membrane is elevated, revealing the structures of the middle ear (Figure 1A). The round window niche is identified, and the bony overhang removed. Next, the stapes is gently palpated to look for a round window reflex to ensure the correct anatomical plane. The window is then punctured for a sample. Optimally, this should be in the inferior portion of the round window to avoid the basilar membrane. The RWM is at a median angle of 113 degrees to the ear canal; thus, a curved sampling device would be needed. As can be seen in the temporal bone specimen, this approach allows access to the basal turn of the cochlea (Figure 1B) [77]. At the conclusion of sampling, a small tissue patch can be applied. The eardrum is then moved back into position.

### 6.4. Progress in the Design of Sampling Devices

Successful perilymph sampling depends on developing a device that safely accesses the scala tympani and atraumatically withdraws a small sample. In humans, sterile glass capillary tubes are commonly used for intraoperative sampling via the RWM with preservation of residual hearing [13,29,30,35,52]. When placed in perilymph, the capillary tube forms a meniscus. This creates a pressure gradient, causing the fluid to move into the tube. The amount of fluid drawn up depends on the radius of the tube, density of the liquid, and surface tension. The angle of approach is also an important factor, as this determines the curvature of the meniscus and thus affects the size of the pressure gradient. Benefits of using the glass capillary tube include a simple methodology and low cost. However, volume aspirated into the capillary can be non-uniform due to variable tube diameter and user technique. In addition, puncture of the RWM with the glass capillary tube can cause CSF outflow into the scala tympani and contamination of the sample [78]. A specific capillary tube has not been validated for intraoperative use in humans; however, multiple research groups have used various sterile glass capillary tubes for perilymph extraction without complication [13,29,30,35,52]. 

Microneedles can also be used for sampling. There are multiple types, most of which have been developed and optimized in animal models. To collect perilymph, the needle is advanced into the RWM and perilymph is drawn up using a syringe. Multiple studies have shown that perforation of the RWM with microneedles does not affect hearing threshold in animals and are generally atraumatic [79,80,81,82]. Microneedles have not yet been tested in humans intraoperatively; however Early et al. recently tested a novel microneedle in fresh frozen human temporal bones. They found that the microneedle with syringe could reliably withdraw 5 µL of perilymph from the scala tympani with minimal contamination and little trauma to the RWM [83]. Although microneedles have a more complex design than the glass capillary tubes, they allow for controlled aspiration of perilymph. This may result in more consistent volumes sampled and may decrease the likelihood of CSF contamination. Using the approach to the round window outlined above and a curved sampling device, articulated instruments that allow incremental advancement of a needle through the round window and subsequent microfluidic withdrawal of 10 µL of perilymph could also be designed (Figure 1C–F). 

### 6.5. Safety and Limitations

Although sampling has been conducted for many years across multiple institutions, there are very few studies directly examining the effects of intraoperative perilymph sampling on post-operative outcomes. Schmitt et al. is the only group to specifically address potential effects on post-CI residual hearing. They compared pre- and post-operative hearing thresholds between patients who underwent CI plus perilymph sampling and randomly selected patients that underwent only CI. No significant differences in residual hearing or speech perception were found between the groups [13]. 

There is a long history of sampling perilymph in stapedectomy patients. This technique has been used previously for profiling perilymph with no apparent complications but, as noted above, is probably not applicable to routine perilymph sampling for sensorineural hearing loss. Some additional insight can be gained from the surgical procedures in which the inner ear is opened. Stapedectomy is considered a safe procedure having only a minimal incidence of SNHL [84]. However, sampling through the stapes footplate would require manipulation of a mobile stapes. Hearing preservation CI in which the ear is not only opened but an implant placed has shown complete hearing preservation rates of 45%, and partial hearing preservation rates of 100% [85,86]. Analysis of cochlear microphonics during implantation suggests that if hearing loss occurs, it is not related to opening the RWM but occurs fairly late in the implantation process [86]. Therefore, a controlled puncture with a sampling of 5–10 μL is unlikely to cause any hearing loss.

There are also some technical limitations of the sampling procedure. Studies in guinea pigs show that perforation of the RWM induces perilymph outflow driven by CSF pressure, leading to possible CSF contamination of the sample, and that samples greater than 10 μL can be significantly contaminated with CSF [87]. There can also be interparticipant variations in perilymph volume, and samples can contain differing amounts of CSF and blood contamination. However, these limitations can be overcome through proper training, sample quality checks, and further optimization of instrumentation such as the microneedles used for extraction. Individual anatomic differences in the cochlear aqueduct must also be considered in sampling methodology. If the cochlear aqueduct is widely patent, there may be excessive perilymph and CSF outflow when the RWM is punctured during CI prior to electrode placement [88]. Only the fluid that first flows out of the RWM is pure perilymph. Therefore, if there is a large volume outflow, the fluid is likely to be contaminated with CSF that has entered the inner ear via the cochlear aqueduct. The likelihood of a CSF gusher is not entirely predictable but has been associated with malformation of inner ear structures, which may be detected on CT [89]. Finally, to move forward with developing this technique, large animal models such as pigs will be needed to test novel devices [90]. 

## 7. Conclusions

New diagnostic techniques are needed to further subclassify SNHL. As proposed here, a round window membrane “tap” can be performed to profile perilymph and determine SNHL subtype and candidacy for adjunctive medical treatment with cochlear implantation. Although perilymph sampling has been performed intraoperatively in humans for decades, it remains controversial due to paucity of literature on post-operative effects on hearing, and further safety studies are needed.
